# Mouse models of fragile X-related disorders

**DOI:** 10.1242/dmm.049485

**Published:** 2023-01-24

**Authors:** Rob Willemsen, R. Frank Kooy

**Affiliations:** Department of Clinical Genetics, Erasmus University Medical Center, 3015 CN Rotterdam, the Netherlands. Department of Medical Genetics, University of Antwerp, 2000 Antwerp, Belgium

**Keywords:** *FMR1*, Fragile X syndrome, Fragile X-associated tremor/ataxia syndrome, Mouse models

## Abstract

The fragile X-related disorders are an important group of hereditary disorders that are caused by expanded CGG repeats in the 5′ untranslated region of the *FMR1* gene or by mutations in the coding sequence of this gene. Two categories of pathological CGG repeats are associated with these disorders, full mutation alleles and shorter premutation alleles. Individuals with full mutation alleles develop fragile X syndrome, which causes autism and intellectual disability, whereas those with premutation alleles, which have shorter CGG expansions, can develop fragile X-associated tremor/ataxia syndrome, a progressive neurodegenerative disease. Thus, fragile X-related disorders can manifest as neurodegenerative or neurodevelopmental disorders, depending on the size of the repeat expansion. Here, we review mouse models of fragile X-related disorders and discuss how they have informed our understanding of neurodegenerative and neurodevelopmental disorders. We also assess the translational value of these models for developing rational targeted therapies for intellectual disability and autism disorders.

## Teaser

**Summary:** We review mouse models of fragile X-related disorders and discuss their advantages, limitations and use as preclinical models, as well as assess the challenges that remain to improve the reproducibility and translational value of these models.

## Introduction

**Fig. 1.**
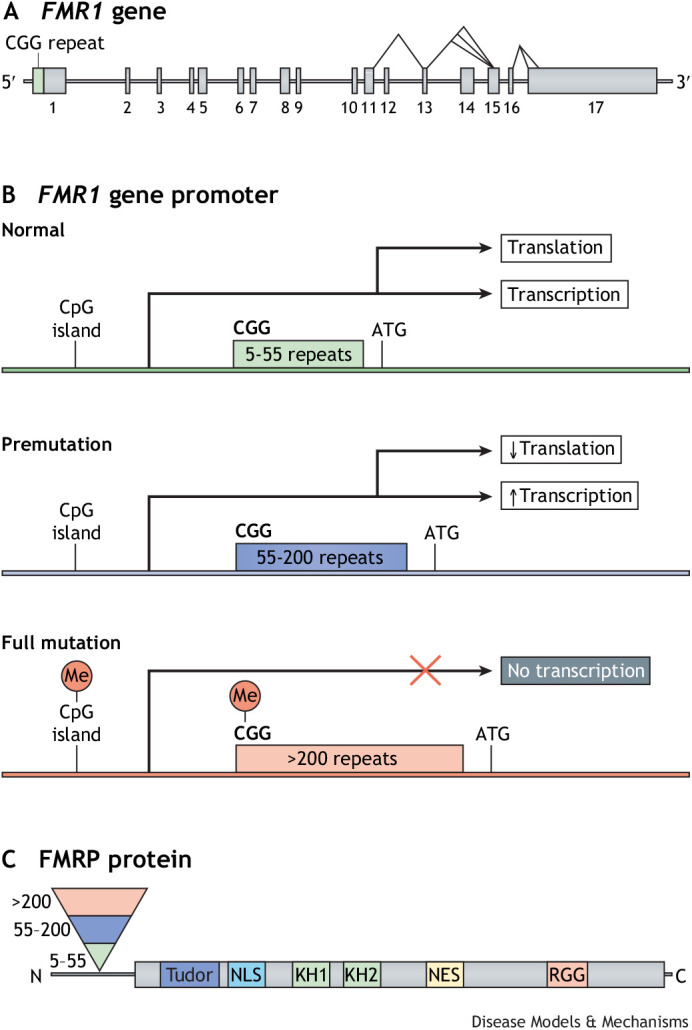
**The structure of the *FMR1* gene and FMRP protein.** (A) Schematic of the human *FMR1* gene showing its potential alternative splicing sites. (B) Schematic showing the transcription and translation of *FMR1* alleles*.* Most individuals carry 5-54 CGG repeats in their *FMR1* gene, which is considered wild-type and is expressed normally (top). Individuals with CGG repeats in the range of 55-200 repeats (shown in blue) carry so-called premutation (PM) alleles, which are associated with elevated *FMR1* mRNA levels and a moderate decrease in FMRP production (middle). Individuals with >200 CGG units repeats (shown in red) carry full-mutation (FM) alleles. In FM, *FMR1* transcription is silenced due to promoter hypermethylation (bottom). (C) The human FMRP protein that is encoded by *FMR1*. Its functional domains include a Tudor methyl-lysine- and methyl-arginine-binding domain, a nuclear localization signal (NLS), two K homology (KH) domains, a nuclear export signal (NES) and an arginine-glycine-rich (RGG) domain.

Fragile X-related disorders are caused by expanded CGG repeats in the 5′ untranslated region of the fragile X messenger ribonucleoprotein 1 (*FMR1*) gene (Fig. 1A) (Herring et al., 2022; Oberlé et al., 1991; Verkerk et al., 1991). In the general population, this repeat is typically 5-55 units long (Fig. 1B) (Nolin et al., 2003), but in individuals with fragile X-related disorders, it is expanded beyond this normal range. Two categories of disease-associated CGG repeats exist: full-mutation (FM) alleles of over 200 CGG repeats, and shorter premutation (PM) alleles of 55-200 repeats (Hagerman et al., 2001; Oberlé et al., 1991; Verkerk et al., 1991) (Fig. 1B).

The inheritance of fragile X-related disorders is best described as X-linked with reduced penetrance in females. The 5′ untranslated region (UTR) CGG repeat in *FMR1* expands upon transmission from generation to generation, predominantly through the maternal line. When its expansion exceeds ∼200 units (FM), it becomes methylated. This inactivates *FMR1* expression and results in fragile X syndrome (FXS) (Fu et al., 1991; Pieretti et al., 1991) (Fig. 1C). It should be noted, however, that FXS can also be caused by deleterious mutations and deletions within *FMR1* (Myrick et al., 2015a; Okray et al., 2015; Zeidler et al., 2021). Clinical hallmarks of FXS include intellectual disability and behavioural abnormalities, such as hyperactivity, anxiety, decreased attention span and various autistiform traits (Hagerman et al., 2017). Males with fragile X show physical characteristics, such as a long face, large everted ears and macroorchidism (see Glossary, Box 1) during and after puberty (Lachiewicz and Dawson, 1994), and ∼20% experience epileptic seizures (Musumeci et al., 1999). Essentially all males with a full mutation are affected, whereas females with FXS have a much wider range of symptoms and can be cognitively unaffected. FXS is one of the most common monogenic disorders. It occurs in all populations in the world with slightly different prevalence rates. A systematic review and meta-analysis estimated its overall prevalence in males at 1:7143 and in females at 1:1111 (Hunter et al., 2014).

**Box 1. Glossary**

**Acoustic startle response:** a rodent's spontaneous shock reaction or startle in response to a controlled pulse of sound.

**Audiogenic seizures:** epileptic attacks induced by exposing the animals to loud (up to 120 db) sounds for a short period, typically around 10 min.

**Automated tube test:** a test that studies the hierarchical relationships between rodents. It consists of two goal boxes connected by a tube that is just big enough for a single mouse to pass. When two mice meet each other in the middle of the tube, one is forced to back down. The one that ends up in the other one's start box is designated the more dominant animal.

**Cerebellar gait ataxia:** abnormal gait characterized by a wide base of support, unsteadiness and irregularity of steps. The walking path of affected individuals typically does not follow a straight line but instead veers in different directions, giving the appearance of stumbling or drunkenness.

**FMRpolyG-positive intranuclear inclusions:** protein aggregates in the nucleus of selected brain cells that contain ubiquitin-positive FMRpolyG. FMRpolyG stands for a polyglycine-containing peptide expressed from non-canonical translation of the 5′ UTR of *FMR1* in FXTAS.

**Intention tremors:** a trembling of a part of the body when attempting a deliberate movement. Intention tremor is specifically associated with cerebellar disease.

**Macroorchidism:** enlarged testicles.

**Mirrored chamber test:** a behavioral test used to measure anxiety in rodents. It involves putting a mouse in a box with a mirror and is based on the assumption that mice show approach-avoidance behavior when confronted by their mirror image.

**Open-field test:** commonly used to measure exploratory behavior and general activity in rodents. It consists of an open field, generally a square, rectangular or circular enclosure, with surrounding walls that prevent escape, for mice to explore.

**Prepulse inhibition of acoustic startle response (PPI):** a phenomenon seen in rodents, in which a weak acoustic auditory stimulus inhibits a subsequent startle response induced by a loud sound.

**Synaptoneurosome:** an *in vitro* preparation to study the synaptic junction. It is a brain cortex homogenate of resealed presynaptic terminals (synaptosomes) still attached to postsynaptic elements (neurosomes).

The current treatments for FXS and fragile X-related disorders are limited and predominantly aimed at treating the symptoms. However, over recent decades, FXS research has become an example of translational research into neurodevelopmental disorders (Bagni et al., 2012; Braat and Kooy, 2014; Hagerman et al., 2017; Santoro et al., 2012; Willemsen and Kooy, 2017). Studies of mouse and other animal models have implicated various signaling pathways and molecules in the pathophysiology of FXS, including metabotropic glutamate receptor 5 (mGluR5, encoded by *Grm5*), γ-aminobutyric acid receptor A (GABA_A_), matrix metallopeptidase 9 (MMP9) and glycogen synthase kinase 3 β (GSK3β, encoded by *Gsk3b*). Although these studies have improved our understanding of the molecular mechanisms underlying FXS, a targeted treatment for FXS has yet to be approved.

Fragile X-associated tremor/ataxia syndrome (FXTAS), a Parkinson-like neurodegenerative disorder, and fragile X-associated premature ovarian insufficiency (FXPOI), a premature ovarian disorder, are caused by unmethylated CGG repeat expansions of 55-200 repeats and are both categorized as PM disorders (Allingham-Hawkins et al., 1999; Hagerman et al., 2001). The prevalence of the PM state among male and female carriers is estimated to be 1:855 and 1:291, respectively (Hunter et al., 2014). Thus, approximately 20 million individuals worldwide are estimated to be carriers of a PM *FMR1* allele. FXTAS occurs in a relatively limited subset of older males with PM alleles (Jacquemont et al., 2004), but female carriers can be affected too (Salcedo-Arellano et al., 2020). Individuals with FXTAS exhibit a range of neuropathologies, including structural brain abnormalities visible by magnetic resonance imaging, Purkinje cell loss, and the presence of ubiquitin- and polyglycine-containing polypeptide (FMRpolyG)-positive intranuclear inclusions (Box 1) in neurons and astrocytes. PM alleles are also associated with FXPOI, which is defined as early entry into menopause. However, this condition lies beyond the scope of this Review and is not discussed further here. For more on FXPOI, we refer readers to a review by Sherman et al. (2014).

Treatments for FXTAS are primarily limited to treating its two major clinical symptoms, intention tremors and cerebellar gait ataxia (Box 1) (Hall et al., 2006). Clinical trials for new FXTAS treatments have produced mixed results. In a double-blind study of the glutamate receptor antagonist memantine, FXTAS patients did not derive benefits (Seritan et al., 2014), whereas two open-label trials using the neurosteroid allopregnalone or the phosphatidylcholine intermediary cyticoline gave promising results but await double-blind follow-up studies (Hall et al., 2020; Wang et al., 2017).

Much of what we have learned about this group of fragile X-related disorders stems from mouse models that have been studied in great detail (reviewed in Berman et al., 2014; Kooy, 2003; Kooy et al., 2017). The aim of this Review is to overview these different FXS and FXTAS mouse models and to discuss their advantages and limitations. We also discuss the use of fragile X mice as preclinical models, and briefly summarize other rodent models of fragile X-related disorders (Box 2).

**Box 2. Rat models of fragile X disorders**

There are no known spontaneous animal models of fragile X disorders, but researchers have developed a range of animal models in addition to the mouse models discussed in this Review. For example, several fragile X rat models have been generated, including a zinc-finger-induced KO model engineered by SAGE Labs (Boyertown, PA, USA) (Asiminas et al., 2019; Engineer et al., 2014; Hamilton et al., 2014) and a CRISPR-induced KO model (Tian et al., 2017). The first rat model lacks 122 bp around the intron/exon boundary of exon 8 of *Fmr1*. Although initially presented as a KO model, this mutant still expresses an *Fmr1* transcript that is missing exon 8, to produce a form of FMRP in which the KH1 domain is located (Fig. 1). The second model has a small deletion in exon 4 of *Fmr1*, inducing a frameshift mutation and the absence of detectable FMRP. Rat models have the advantage of being larger in size than mice, which facilitates anatomical studies, and generally perform better in learning tasks (Szpirer, 2020). This, however, comes at the expanse of less powerful genetic tools. The cellular and neurological phenotypes of these KO rats were similar to those of KO mice (Berzhanskaya et al., 2016, 2017; Kulkarni and Sevilimedu, 2020; Tian et al., 2017).

## The fragile X gene

**Fig. 2.**
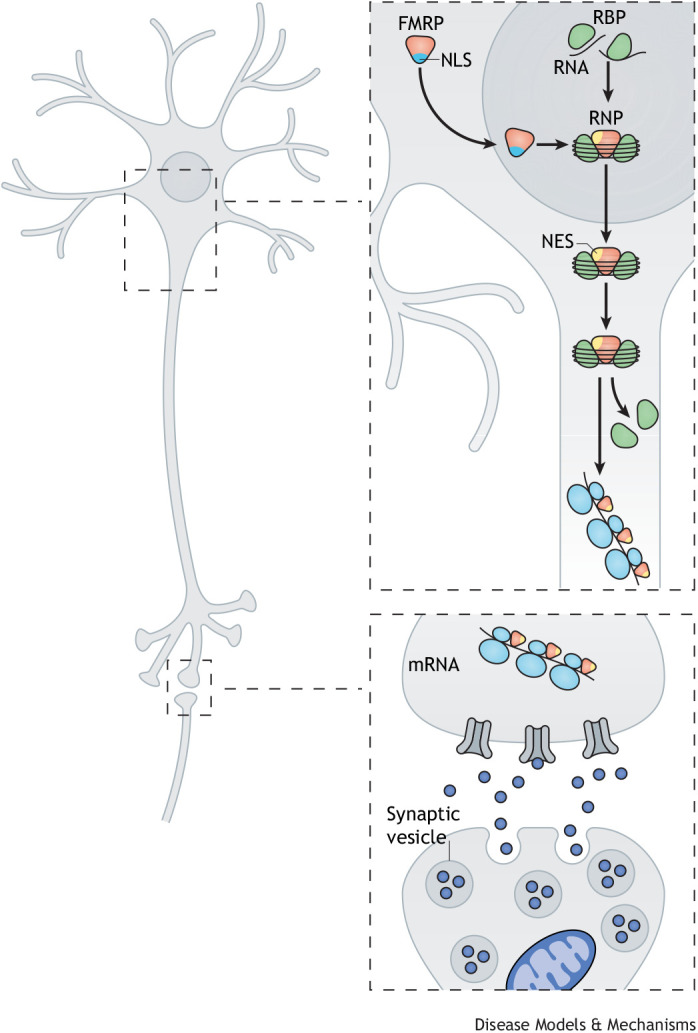
**FMRP function in neurons.** FMRP is synthesized in the cytoplasm and enters the nucleus via its nuclear localization signal (NLS). In the nucleus, FMRP binds to target mRNAs and other proteins, forming a ribonucleoprotein (RNP) particle. The FMRP-RNP particle is transported back to the cytoplasm via the nuclear export signal (NES) of FMRP. FMRP-RNP particles regulate protein synthesis in the cytoplasm of a neuron. Some FMRP-RNP particles are also packed into mRNA-granules and transported into the dendrites of the neuron. During transport, FMRP acts as a translational repressor of the target mRNAs within the granules. Upon synaptic stimulation of group I mGluRs, FMRP allows the translation of its mRNA targets. The translated proteins are involved in the cyclic internalization of AMPA receptors and other neuronal processes. RBP, RNA-binding protein.

The *FMR1* gene is located on human chromosome X q27.3 and is transcribed into 17 exons. It is well conserved throughout evolution (Eichler et al., 1993) and encodes FMRP, which is involved in many cellular processes through its functional domains. These include two (potentially three) K homology (KH) domains and an arginine-glycine-glycine (RGG) box, both of which are RNA-binding domains, as well as protein-interaction domains (Fig. 1C). It also has a nuclear localization signal (NLS) and nuclear export signal (NES), which drive the shuttling of FMRP between the cytoplasm and nucleus (Devys et al., 1993; Eberhart et al., 1996; Feng et al., 1997a; Willemsen et al., 1996). At the N-terminus, a tandem domain called Agenet (also known as Tudor) is potentially involved in the binding of trimethylated lysines (Fig. 1C) (He and Ge, 2017; Maurer-Stroh et al., 2003; Myrick et al., 2015b). *FMR1* gene expression is widespread; it is expressed in spermatogonia and is abundantly expressed in neurons (Bakker et al., 2000; De Diego Otero et al., 2002; Devys et al., 1993; Feng et al., 1997a; Tamanini et al., 1997). The subcellular distribution of FMRP is largely cytoplasmic. High concentrations of FMRP are found associated with ribosomes attached to the endoplasmic reticulum, with free ribosomes in the cytoplasm, at the bases of neuronal dendrites and within dendritic spines (Bakker et al., 2000; Feng et al., 1997b; Weiler et al., 1997; Willemsen et al., 1996). In mice, *Fmr1* expression is activated in early embryonic development and is high in all embryonic tissues. In successive stages of embryonic development, its expression diminishes, and in adult mice, it shows tissue-specific expression (Hinds et al., 1993). In neurons, FMRP transports RNA and protein cargoes to the synapses where they facilitate local translation. FMRP is also involved in RNA stability, splicing, editing and interference (reviewed in Bagni and Zukin, 2019; Richter and Zhao, 2021; Richter et al., 2015) (Fig. 2).

**Fig. 3.**
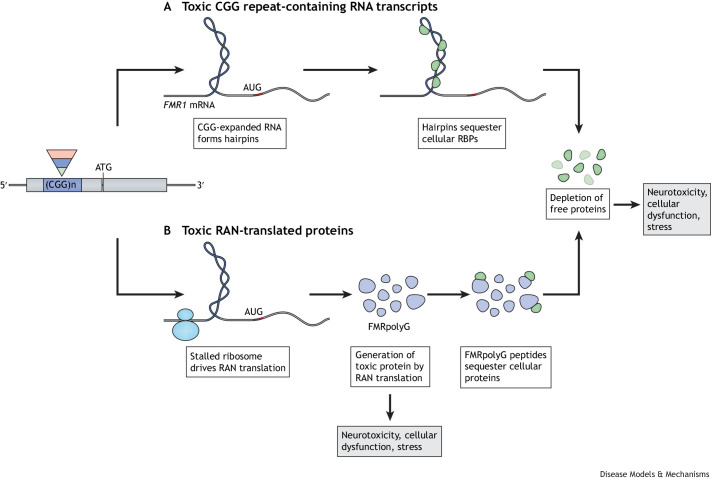
**Two models of expanded CGG repeat toxicity.** (A) The CGG repeat-mediated RNA toxicity and sequestration model assumes that RNA-binding proteins (RBPs) are sequestered through their interactions with the expanded CGG repeat-containing *FMR1* mRNA. These proteins in turn recruit other essential cellular proteins and sequester them, such that these essential proteins are unavailable for their normal cellular functions. (B) The toxic polypeptide model assumes that the ribosome translation initiation complex stalls near the CGG repeat hairpin formed on the *FMR1* mRNA. This drives the repeat-associated non-AUG (RAN) translation of *FMR1* mRNA using a nearby AUG start site. This results in a frame shift and the production of the polyglycine-containing polypeptide (FMRpolyG), among other polypeptides, which interfere with normal cellular function via an unknown toxic mechanism.

Carriers of PM alleles, with or without FXTAS, have elevated levels of *FMR1* mRNA and slightly reduced levels of FMRP translation (Tassone et al., 2000). The pathophysiology of the neurodegenerative aspects of FXTAS is poorly understood, with two prevailing theories seeking to explain it (Fig. 3) (Boivin et al., 2018; Sellier et al., 2017; Todd et al., 2013). According to the first theory, the abundant RNAs containing the expanded repeats sequester other proteins in the cell, leading to cellular dysfunction, neurotoxicity, cellular stress and disruption of cellular homeostasis (Fig. 3A) (He et al., 2014; Sellier et al., 2010, 2013; Sofola et al., 2007). The second theory stipulates that the non-canonical translation across the repeat results in the production of polyglycine-, polyalanine- and polyglutamine-containing proteins that themselves have toxic effects in the cell or that sequester essential cellular proteins, reducing their availability and therefore causing cellular dysfunction (Fig. 3B) (Todd et al., 2013). This non-canonical mechanism of microsatellite repeat expansion translation has been named repeat associated non-AUG (RAN) translation. RAN translation has, for the first time, also been described in myotonic dystrophy 1 (Zu et al., 2011). However, it cannot be excluded that a combination of both theorized mechanisms is involved in the pathogenesis of FXTAS.

To further our understanding of the pathophysiology of FXS and FXTAS, various animal models have been generated, including mouse models of FXS and FXTAS. Each of these models has contributed to our understanding of the molecular, cellular, physiological and behavioral deficits associated with the fragile X-related disorders. Here, we focus on two main types of mouse model, knock-out (KO) and repeat expansion models. KO models recapitulate FXS, as the *Fmr1* gene is not expressed in these animals, whereas repeat expansion models are used to model FXTAS and repeat instability. For a summary of all of the KO, conditional and cell type-specific mouse models of FXS and FXTAS that have been described in the literature, see Table 1. For more information on non-murine models of FXS and FXTAS, we direct readers to the extensive reviews by Drozd et al. (2018), Kooy et al. (2017) and Curnow and Wang (2022).

**Table 1.**
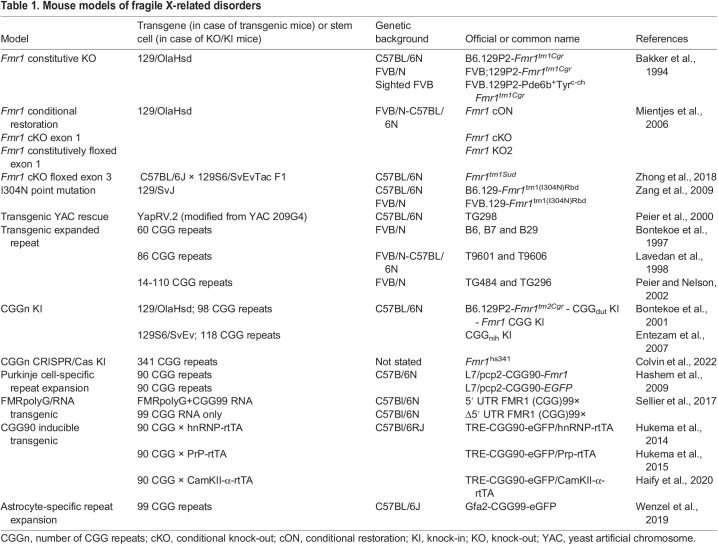
Mouse models of fragile X-related disorders

## KO mouse models of FXS

The first mouse model of FXS, which is still in use today, is a KO model (Bakker et al., 1994), in which the deletion of exon 5 interrupts the coding sequence. Although small quantities of mutated RNA are transcribed in this model, no protein is produced. Following this constitutive KO model, a conditional *Fmr1* KO (cKO) model was generated to enable researchers to inactivate the gene in a spatial and temporal manner (Mientjes et al., 2006). In this model, exon 1 is flanked by two loxP sites, which recombine in the presence of Cre recombinase to remove this exon, resulting in the complete absence of *Fmr1* transcription. As Cre can be expressed under the control of tissue-specific or doxycycline-dependent promoters, the excision of exon 1 can be induced in certain tissues or at certain time points (Lovelace et al., 2020). A conditional restoration model has also been generated, in which *Fmr1* can be reactivated upon the Cre­mediated deletion of an inserted neomycin gene (Guo et al., 2011).

A second constitutive KO model has been generated by crossing the cKO mouse with a transgenic strain that carries a CAG-Cre expressor that directs Cre expression from the CAG promoter in mature oocytes. In this model, exon 1 is non-reversibly excised (Mientjes et al., 2006). One of the advantages of this ‘KO2’ model over the original KO is that it does not express mutated RNA nor the neomycin marker, which is present in the targeting construct of the first-generation original *Fmr1* KO mouse model. Although these two constitutive KO models remain to be systematically compared, no major differences have been reported between them and both are used interchangeably in various studies.

Another cKO model contains loxP sites flanking exon 3 of *Fmr1*, the removal of which results in the complete loss of transcription. To date, this model has only been described in a single study, which reported the importance of retinoic acid signaling for the homeostatic regulation of synaptic transmission at inhibitory synapses (Zhong et al., 2018). Apart from KO models, researchers have also developed models that carry mutations in *Fmr1.* One such model contains a point mutation that aims to recapitulate a human *FMR1* mutation, I304N, which is associated with an extremely severe clinical FXS presentation (De Boulle et al., 1993). This model was generated by replacing a large part of *Fmr1* with an I304N-mutated exon 10 using a self-excising loxP cassette in 129/SvJ embryonic stem cells (Zang et al., 2009). Thus, several constitutive as well as conditional FXS models have been generated. In the next section, we discuss the different phenotypes of these various mouse FXS models.

## FXS KO model phenotypes

### Craniofacial and anatomical phenotypes

A hallmark of FXS patients is their facial features, which include a long face, tall forehead, prominent jaw and large, everted ears (Hagerman et al., 2017). Micro-computed tomography images have shown that the *Fmr1* KO mouse model has altered mandibles and altered outer and inner skull dimensions relative to wild-type mice (Heulens et al., 2013). However, due to the differences in skull shapes between mice and humans, it is difficult to conclude the extent to which these anatomical features of *Fmr1* KO mice resemble the facial features of FXS patients. Macroorchidism, a clinical feature of post-pubertal boys with FXS, is also consistently recapitulated in both constitutive and conditional KO mouse models (Bakker et al., 1994; Kooy et al., 1996; Mientjes et al., 2006). FXS patients also have a larger caudate nucleus and a smaller posterior cerebellar vermis, amygdala and superior temporal gyrus relative to non-FXS individuals, as well as abnormal cortical lobe volumes (Gothelf et al., 2008). Brain regions in *Fmr1* KO mouse models appear to be normal in terms of their size and shape, although these mice do have a relatively larger white-matter volume in major structures throughout the brain and in areas associated with frontostriatal circuitry. These phenotypes are seen when *Fmr1* KO mouse models are bred on the FVB/N background, but not on the C57BL6/J background (Ellegood et al., 2010; Kooy et al., 1999; Lai et al., 2016). Thus, the neuroanatomy of these KO models appears to differ from that of FXS patients, although a more systematic comparison of the neuroanatomy of both species is yet to be performed. In the next section, we discuss the cellular and molecular pathways that underlie the abnormal neuroanatomy of these models.

### Cellular and molecular phenotypes

FXS patients have been reported to have a higher density of cortical dendritic spines that have an immature morphology (Irwin et al., 2002). FXS KO mice also have submicroscopic neuroanatomical abnormalities, which are brain region and age dependent, including dendritic spines that are immature in their appearance, evidenced by the overrepresentation of both elongated, thin spines and short, cup-shaped ones (Speranza et al., 2022; Wijetunge et al., 2014).

Similar to FXS patients, *Fmr1* KO mice do not produce Fmrp (Bakker et al., 1994). Fmrp associates with specific dendritic mRNAs as well as with the polyribosomes responsible for protein translation, and is believed to function as a negative regulator of mRNA translation (Fig. 2) (Laggerbauer et al., 2001). Indeed, *Fmr1* KO mice show increased protein synthesis in total brain homogenates and in synaptoneurosomes (Box 1) (Muddashetty et al., 2007; Dölen et al., 2007). These observations suggest a role for Fmrp in synaptic plasticity in mice. Indeed, synaptic transmission is altered in *Fmr1* KO mice (Pfeiffer and Huber, 2007). The long-term potentiation (LTP) and long-term depression (LTD) of excitatory synaptic transmission are key cellular mechanisms in learning and memory (Kandel, 2001). α-amino-3-hydroxy-5-methyl-4-isoxazolepropionic acid (AMPA) receptors at the postsynaptic membrane are involved in the modulation of synaptic transmission (Kandel, 2001). LTP induces an increase in AMPA receptor numbers and synaptic strengthening, whereas LTD decreases AMPA receptor numbers. Both *Fmr1* KO mouse models have a reduced LTP in prefrontal and cingulate cortexes of adult animals relative to wild-type ones (Koga et al., 2015), but not in KO mice younger than 12 months (Martin et al., 2016). LTP abnormalities in other brain regions have not been reported in these mice (Godfraind et al., 1996).

LTD can be induced by the activation of group 1 mGluRs (which play a role in glutamate-induced synaptic plasticity) in a form of LTD that is protein-synthesis dependent. Remarkably, *Fmr1* KO mice show reduced mGluR group 1-dependent LTD (Huber et al., 2001). Additional downstream signaling pathways connect mGluR signaling to FMRP-regulated mRNA translation. These signaling pathways include the extracellular signal-regulated kinase (ERK) pathway and the mammalian target of rapamycin (mTOR) pathway, both of which are deregulated in FXS (Huber et al., 2001; Sharma et al., 2010; Hoeffer et al., 2012; Wang et al., 2012). Lack of FMRP expression also results in the upregulation of a subset of mRNAs at the synapse, including mRNAs for GSK3, striatum-enriched protein tyrosine phosphatase (STEP, encoded by *PTPN5*), MMP9, amyloid precursor protein (APP), the small GTPase Ras family and phosphoinositide 3-kinases (PI3Ks) (Hu et al., 2008; Stornetta and Zhu, 2011; Lim et al., 2014). The altered expression patterns of these genes suggest the involvement of these pathways in FXS. The exact roles of these pathways remain to be elucidated. An important role for the inhibitory GABAergic and dopaminergic systems has also been proposed in the etiology of FXS. The reduced expression of several GABA-receptor subunits and of dopamine receptors have been reported in *Fmr1* KO mice (D'Hulst et al., 2006, 2009; Gantois et al., 2006; Sørensen et al., 2015; Zupan and Toth, 2008; Zupan et al., 2016). How these cellular and molecular phenotypes result in deficits in cognition and behavior is discussed in the following section.

### Cognitive and behavioral phenotypes

FXS KO mouse models show both cognitive and behavioral changes, some of which recapitulate those seen in FXS patients. Hyperactivity is a consistent feature in pre-pubertal boys with FXS and is seen in constitutive FXS KO models. However, the extent of the hyperactivity of these mice models differs between studies and ranges from a mild and marginally significant increase to an almost doubling of activity in KO mice relative to control littermates (for example, compare studies by Cogram et al., 2019 and Kooy et al., 1996). The original constitutive FXS KO model is also reported to exhibit impaired inhibitory control and disrupted olfactory processing relative to wild-type mice (Larson et al., 2008).

Cognitive functioning in mice is assessed using a variety of tests, such as the Morris water maze test, in which mice need to find a hidden platform in a pool using indirect visual cues. The original constitutive FXS KO mouse model exhibited deficits in this test, most notably in the reversal phase of the test, when the platform was relocated to the opposite side of the pool (Bakker et al., 1994; D'Hooge et al., 1997). However, these results were not always reproduced by other laboratories (Dobkin et al., 2000; Paradee et al., 1999). We now know that these differences between these studies might be due to the use of KO mice of different genetic backgrounds. The first experiments in which a learning deficit in the Morris water maze test was reported were performed using KO mice bred onto a mixed genetic background, 129P2 and C57BL/6 (Dobkin et al., 2000; Paradee et al., 1999). When the KO mice were bred onto a C57BL/6 background, however, the same deficit was not reported (Paradee et al., 1999). In the same study, the authors demonstrated that KO mice bred onto a hybrid C57BL6/129 genetic background exhibited the same deficits in the Morris water maze test that were reported in the original studies by Bakker et al. (1994) and D'Hooge et al. (1997).

Spontaneous epileptic seizures occur in individuals with FXS but are only rarely observed in FXS KO models (Armstrong et al., 2022). These seizures can be induced in KO mice using audiogenic stimuli (Musumeci et al., 2007). FVB/N mice are more sensitive to audiogenic seizures (Box 1) than are C57BL/6J mice (Kasugai et al., 2007), as seen in the seizure preponderance of *Fmr1* KO mice bred on the FVB/N background (Heulens et al., 2012; Musumeci et al., 1999). The increased susceptibility of this strain to the audiogenic induction of seizures might resemble the increased incidence of children and adolescents with FXS (Berry-Kravis, 2002; Van der Aa and Kooy, 2020). Enhanced responses to audiogenic stimuli have been observed in the electrophysiological recordings of some brain regions of *Fmr1* KO mice (Nguyen et al., 2020). Differences in the acoustic startle response and in the prepulse inhibition (PPI) of acoustic startle response (Box 1) have also been reported in constitutive KO models (Chen and Toth, 2001; Errijgers et al., 2008; Kazdoba et al., 2014; McCullagh et al., 2020). Interestingly, a decreased PPI response is reported to be a hallmark of FXS patients (Frankland et al., 2004; Hessl et al., 2009).

Increased anxiety has also been reported in the mirrored chamber test (Box 1), in which *Fmr1* KO mice show a greater aversion to the central mirrored chamber (Spencer et al., 2005). However, in the open-field test (Box 1), constitutive *Fmr1* KO mice tend to spend more time in the center of the box, indicative of a decrease in anxiety. Other social interaction tests have been performed on *Fmr1* KO mice with variable results. However, the automated tube test (Box 1) has unambiguously shown that *Fmr1* KO mice are socially dominant, winning almost any fight in the tube when confronted with a wild-type littermate (de Esch et al., 2015).

Unexpectedly, the phenotypes of mice with an Fmrp I304N point mutation, which causes a clinically severe FXS in humans that includes profound intellectual disability and excessive macroorchidism (De Boulle et al., 1993), resemble those of *Fmr1* KO mice; for example, in their degree of macroorchidism, in their behavioral responses and in their synaptic electrophysiological measurements (Zang et al., 2009). This was a surprise, as researchers expected a much more severe phenotype in Fmrp^I304N^ mice given the severe clinical presentation in humans. The reason for this discrepancy has never been properly resolved, but lower-than-expected expression levels of the mutated *Fmr1* might have played a role (Zang et al., 2009). It is also possible that an additional mutation in the phosphorylase kinase regulatory subunit α 2 (*PHKA2*) gene, which is present in an FMRP^I304N^ patient (De Boulle et al., 1993) but not in the mouse model, contributes to the difference in disease severity seen in the mouse model relative to the individual with the mutation. It should be noted, however, that the *PHKA2* mutation itself is responsible for a usually mild form of liver glycogenosis (Fernandes et al., 2020).

Thus, the constitutive FXS KO models resemble FXS patients to a certain extent, i.e. they both exhibit macroorchidism, learning deficits and decreased PPI. Definitive evidence that these shared characteristics can be attributed only to the absence of *Fmr1* in these mice was provided by the generation of transgenic overexpression models and crossing these with KO mice, as we discuss next.

## Transgenic overexpression models

An overexpression *Fmr1* model was first generated in 2000 by introducing a yeast artificial chromosome (YAC) containing the human *FMR1* gene with ∼20 CGG repeats into mouse embryonic stem cells (Peier et al., 2000). The resulting line, TG298, overexpresses *FMR1* mRNA and FMRP at levels at least ten times that of the endogenous Fmrp. In contrast to the macroorchidism observed in FXS KO mouse models, transgenic TG298 mice had smaller testicles compared to their wild-type littermates. They also displayed reduced anxiety-related responses with increased exploratory behavior, unlike those of *Fmr1* KO mice. The transgenic mice were also intercrossed with an *Fmr1* KO mouse, which led to the rescue or even ‘overcorrection’ of the anatomical and behavioral abnormalities observed in the KO model (Peier et al., 2000). For example, mice obtained by crossing the transgenic TG298 line with the *Fmr1* KO mice had testicular weights that were indistinguishable from controls, and the behavioral abnormalities characteristic of KO mice, including increased locomotor activity and decreased anxiety-related responses, were ameliorated. These experiments thus strongly indicate that the abnormalities observed in the FXS KO model can be rescued by the expression of human FMRP and that inactivation of *Fmr1* underlies the abnormalities of the KO mouse model. Importantly, these studies also demonstrate that *Fmr1* overexpression can have adverse consequences, such as an increased acoustic startle response, highlighting the notion that FMRP levels need to be tightly controlled.

## PM mouse models of FXTAS

Interestingly, mouse models of the *Fmr1* PM were available before the first clinical reports of FXTAS in carriers of PM alleles (Bontekoe et al., 2001; Lavedan et al., 1998). *Fmr1* PM transgenic mice were initially developed to study the timing and molecular mechanisms of CGG repeat instability (Bontekoe et al., 2001; Lavedan et al., 1998). They were generated to express expanded CGG repeats in the genome but did not recapitulate the CGG repeat instability seen in humans with a repeat expansion (Bontekoe et al., 2001; Lavedan et al., 1998). Even YAC-bearing transgenic mice that contained a PM-sized CGG repeat expansion (50-200 CGG repeats) in the 5′ region of the entire human *FMR1* did not recapitulate human transgenerational repeat instability (Peier and Nelson, 2002). In a 2001 study, a targeting construct containing 98 CGG repeats was knocked into *Fmr1* to generate the CGG_dut_ knock-in (KI) mouse model (Bontekoe et al., 2001). This mouse model showed moderate CGG repeat instability upon both paternal and maternal transmission. Unfortunately, however, when the CGG repeat expansions reached over 200 in the progeny of CGG_dut_ mice, this did not result in the methylation of the *Fmr1* promoter region, as seen in humans. In a similar strategy, a targeting construct containing 118 CGG repeats was introduced into mice (Entezam et al., 2007). The resulting CGG_nih_ KI mouse model showed moderate repeat instability, but also exhibited a lack of *Fmr1* promoter methylation when the repeat was expanded into a FM-sized allele. Most recently, the largest PM allele so far was cloned into the *Fmr1* locus using CRISPR/Cas technology (Colvin et al., 2022). The resulting *Fmr1*^hs341^ mice had an expansion of 341 CGG repeats obtained from a human patient. Yet, even expansions of this size, which are well within the FM range in humans, did not result in promoter methylation. Thus, the methylation trigger appears to differ between humans and mice.

### FXTAS KI model phenotypes

The discovery of FXTAS revived interest in the PM mouse models, given that these KI mice might provide crucial insights into the molecular mechanisms that underlie FXTAS with aging. Both KI models, CGG_dut_ and CGG_nih_, showed elevated *Fmr1* mRNA levels, reduced Fmrp production, the presence of ubiquitin-positive intranuclear inclusions throughout the brain and specific behavioral deficits, including late-onset ataxia, memory impairment and impaired motor performance, all key features of FXTAS (Entezam et al., 2007; Willemsen et al., 2003; reviewed in Foote et al., 2016). Importantly, RAN translation occurs in CGG_dut_ KI mice, together with the accumulation of FMRpolyG-positive intranuclear neuronal inclusions (Fig. 3B), whereas CGG_nih_ KI mice lack RAN translation products.

This discrepancy is due to a minor difference in the design of these two mouse models. Despite the high levels of concordance between the mouse *Fmr1* and human *FMR1* sequences, one crucial difference between the species is the presence of a TAA stop codon immediately upstream of the CGG repeat in the native murine sequence, which is absent from the human sequence (Rodriguez et al., 2020). In wild-type mice and in the CGG_nih_ KI model, which contains the murine sequence 5′ of the CGG repeat, this TAA stop codon prevents the translation of FMRpolyG from the repeat. However, in the CGG_dut_ KI mouse model, this TAA stop codon is absent because a human sequence was used here as the cloning vehicle, explaining why RAN translation was observed in this model. Importantly, histopathological and behavioral analyses of both the KI mouse models have revealed reduced levels of intranuclear inclusions and of pronounced behavioral deficits in the CGG_nih_ mouse model relative to the CGG_dut_ mouse model.

In summary, the development of both KI mouse models has facilitated studies into the underlying basis of CGG repeat instability and the role of RNA toxicity in the neuropathology in FXTAS. They have also provided crucial information about the molecular changes that take place at the onset and during the progression of FXTAS. In addition to these KI mouse models of FXTAS, inducible cell- and tissue-specific PM mouse models have been generated, which we discuss in the next section.

## Inducible and cell- and tissue-specific FXTAS models

To investigate the two main hypotheses that seek to explain the underlying molecular basis of FXTAS, namely, RNA toxicity versus FMRpolyG toxicity (Fig. 3), two transgenic mice with CGG repeat sizes in the PM range have been generated. The first of these models, called CMV-Cre/5′-UTR-99×CGG-GFP, carries the entire 5′ UTR of human *FMR1*, including 99 CGG repeats. This model expresses CGG RNA and produces FMRpolyG (Sellier et al., 2017). The second model, called CMV-Cre/Δ5′-UTR-99×CGG-GFP, also contains 99 CGG repeats but lacks the non-canonical ACG start codon. This model only expresses CGG RNA and does not produce RAN translation products (Sellier et al., 2017), but is otherwise identical to the CMV-Cre/5′-UTR-99×CGG-GFP model. The first model, in which FMRpolyG is translated, develops FXTAS-like phenotypes, including FMRpolyG-positive intranuclear inclusions and neuronal cell death. In contrast, the second model is phenotypically indistinguishable from control mice. This suggests that RAN translation of the expanded CGG repeat into FMRpolyG, but not the expansion of the CGG repeat, drives pathogenesis in FXTAS.

Tissue-specific PM mouse models, such as the Purkinje cell-specific mouse model, allow researchers to study the role of an ectopically expressed, expanded CGG repeat in the pathogenesis of FXTAS (Hashem et al., 2009). In this model, the presence of ubiquitin-positive inclusions in Purkinje cells impaired motor performance. Purkinje cell loss was also observed, indicating a role for CGG repeat mRNA expression in neurodegeneration. The concept of RAN translation was not known at the time of publication of this model, but as the 90-repeat PM was derived from the human *FMR1* sequence, it is plausible that the TAA stop codon preceding the murine CGG repeat was replaced and, therefore, RAN translation is present in this model.

Although these mouse models have significantly contributed to our understanding of the pathogenesis of FXTAS, they cannot be used to optimize the timing of therapeutic interventions; this requires inducible transgene expression to assess whether switching off the transgene would slow or reverse the disease process. To address this limitation, doxycycline-inducible double transgenic mouse models have been developed using the Tet-on system in a transgenic line that expresses 90 CGG repeats. Several transgenic driver lines have been used to drive the expression of this expanded repeat RNA in a ubiquitous or cell- or tissue-specific manner. These driver lines include the heterogeneous nuclear ribonucleoprotein-reverse tetracycline transactivator (hnRNP-rtTA), prion protein-reverse tetracycline transactivator (PrP-rtTA) and Ca^2+^/calmodulin-dependent protein kinase IIA-reverse tetracycline transactivator (CamKII-α-rtTA) (Haify et al., 2020; Hukema et al., 2014, 2015).

The characterization of these inducible mouse models has provided proof for disease reversibility, especially when transgene expression was halted at an early stage of FXTAS. In addition, observations in these inducible mouse models indicate that behavioral phenotypes do not correlate with the presence of FMRpolyG inclusions (Castro et al., 2017; Haify et al., 2021). These mouse models have also been used to develop various strategies for targeted therapeutic intervention. For instance, the hnRNP-rtTA inducible model showed the potential for a designer RNA-binding probe {9-hydroxy-5,11-dimethyl-2-[2-(piperidin-1-yl)ethyl]-6H-pyrido(4,3-b)carbazol-2-ium} to reduce FMRpolyG-mediated toxicity. Small molecule 1a, as this compound was named by its inventors (Disney et al., 2012), reduced the number of intranuclear inclusions in cultured primary neurons from hnRNP-rtTA mice (Haify et al., 2021). As an RNA-binding probe, small molecule 1a shields the CGG repeat by binding GG-mismatch binding spots, preventing both the binding of ribonuclear proteins to the repeat as well as RAN translation (Disney et al., 2012).

Finally, to study the role of astrocytes in FXTAS, a PM transgenic mouse line has been generated that expresses a 99-repeat CGG segment fused to a gene encoding a fluorescent protein in astrocytes throughout the brain using the astrocyte-specific *Gfa2* promoter (Wenzel et al., 2019). This model recapitulates the key features of FXTAS, including ubiquitin- and FMRpolyG-containing intranuclear inclusions in astroglia and neurons, as well as motor function deficits. Interestingly, these mice showed a prion-like spread of the inclusion pathology from astrocytes to neurons by a cell-to-cell transfer mechanism (Wenzel et al., 2019). Thus, the PM models have helped us discriminate the role of RAN translation from that of repeat expansion and have contributed to our understanding of the role of specific cell types in the pathogenesis of FXTAS. Moreover, the inducible mouse models provided evidence for the reversibility of the FXTAS clinical symptoms and thus raise hope for treatment, as drugs have proven efficacious even if administered after the onset of FXTAS. This is not to say that these models do not have their limitations, however.

## Limitations of FXS mouse models

**Fig. 4.**
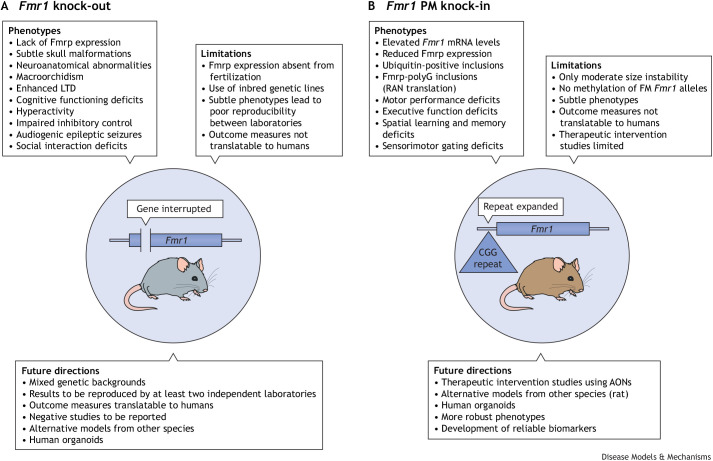
**Summary of FXS mouse models.** (A) The general phenotypes, limitations and future research directions for FXS knock-out mouse models, which do not express FMRP and are therefore aimed at recapitulating the full mutation in patients. (B) The phenotypes, limitations and future research directions for the FXTAS knock-in mouse model. AONs, antisense oligonucleotides; FM, full mutation; *Fmr1*, fragile X messenger ribonucleoprotein 1 gene; Fmrp, fragile X messenger ribonucleoprotein; LTD, long-term depression; PM, premutation; RAN translation, repeat associated non-AUG translation.

Despite the evolutionary similarities between mice and humans, fundamental differences exist between the mouse models and fragile X disorders in humans. In constitutive *Fmr1* KO mice, the *Fmr1* gene is absent from fertilization as it is permanently inactivated (Fig. 4A). As a consequence, individuals with an FM allele differ from constitutive KO mouse models in having FMRP translated and functioning during the first few weeks of development (Zhou et al., 2019). The consequences of this developmental difference are entirely unknown. Moreover, the embryonic stem cells used to generate the original KO model were derived from the 129P2 mouse strain. Due to the poor breeding and behavioral characteristics of this strain, mutants were backcrossed onto other strains, including the C57BL/6, FVB or sighted FVB strains. The C57BL/6 strain is the most commonly used and suitable for most murine experiments (Võikar et al., 2001). Mice of the FVB strain breed even more prolifically and are therefore preferred by some institutions. This albino strain, however, has the disadvantage of becoming virtually blind before adolescence due to retinal degeneration (Taketo et al., 1991). The sighted FVB strain is a pigmented version of the FVB strain that does not suffer from retinal degeneration (Errijgers et al., 2007). The differing genetic backgrounds further complicate the interpretation of results obtained from KO mouse model studies.

Repeat expansion KI models have also been generated but, unlike in humans, these expanded repeats are never methylated in mice, not even when the repeat size far exceeds the methylation threshold in humans. Thus, these KI mice cannot be used to model FXS (Brouwer et al., 2007; Colvin et al., 2022). The reason for this species-dependent difference in methylation is unknown. As PM alleles are never methylated, repeat expansion models are valid for modeling FXTAS (Fig. 4B). However, developing PM models is complicated by a minor but fundamental difference between the murine and human sequence. As discussed above, a TAA stop codon is present in the murine sequence just upstream of the repeat, preventing the translation of the polyG polypeptide that is the most common product of RAN translation in man (Berman et al., 2014). Models with engineered repeat expansions of the murine sequence, such as the CGG_nih_ KI mouse, are thus devoid of the primary RAN translation product and only partially mimic FXTAS (Entezam et al., 2007). The CGG_dut_ KI mouse, which carries the human repeat expansion and thus has RAN translation, has additional subtle differences to the CGG_nih_ KI model (Bontekoe et al., 2001). The pair of transgenic models developed by Sellier et al. (2017), one with and one without the TAA stop codon, were meant to investigate the separate roles of RAN translation and toxic repeat transcripts in FXTAS. However useful these models are, the repeat expansion in these models was engineered in a *FMR1* promoter fused to a reporter gene, thus lacking the coding *FMR1* sequence. Therefore, the influence of aberrantly expressed *FMR1* on the phenotype of this model, if any, could not be determined.

Attempts to fully correct FMRP expression in the KO mice have failed. Overexpression constructs for these experiments contained human *FMR1* in YACs, which generated an excess of human *FMR1* RNA and protein. Crossing these *FMR1*-overexpressing mice with KO animals caused behavioral abnormalities that were in part opposite to the abnormalities observed in the KO mice (Peier et al., 2000). Thus, however useful, each of the many murine models of fragile X-related disorders has disadvantages. Perhaps other rodent models could compensate for some of the murine models' limitations. Although *Fmr1* KO rats have been generated (see Box 2), a rat PM model has not, and so we do not know if such a model has a different methylation threshold and would therefore recapitulate the human transcriptional deregulation.

## Preclinical studies in FXS mouse models

**Fig. 5.**
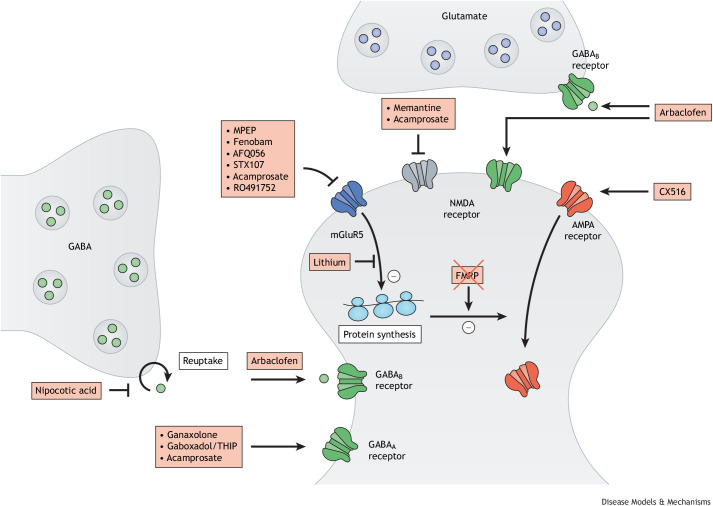
**Pathways involved in fragile X syndrome and synaptic targets of therapeutic interventions.** Several types of drugs can interact with neuronal receptors, which might rescue the disturbed synaptic transmission in FXS. These include NMDA, AMPA, mGluR5, GABA and muscarinic receptors. MPEP, 2-methyl-6-(phenylethynyl)pyridine; THIP, 4,5,6,7-tetrahydroisoxazolo(5,4-c)pyridin-3-ol.

Mouse models have provided insights into the neurobiological mechanisms of fragile X-related disorders and have enabled the identification of drug targets. In fact, many of the pathways that are now known to be compromised in FXS were discovered in these mouse models. Based on this knowledge, drugs have been selected to target the molecular deficits in FXS mouse models in preclinical studies (Fig. 5). In fact, many compounds have been shown to have the potential to rescue the susceptibility to seizures, the neuronal spine abnormalities and the behavior of *Fmr1* KO mice. A comprehensive overview of these studies is provided in Table 2.

**Table 2.**
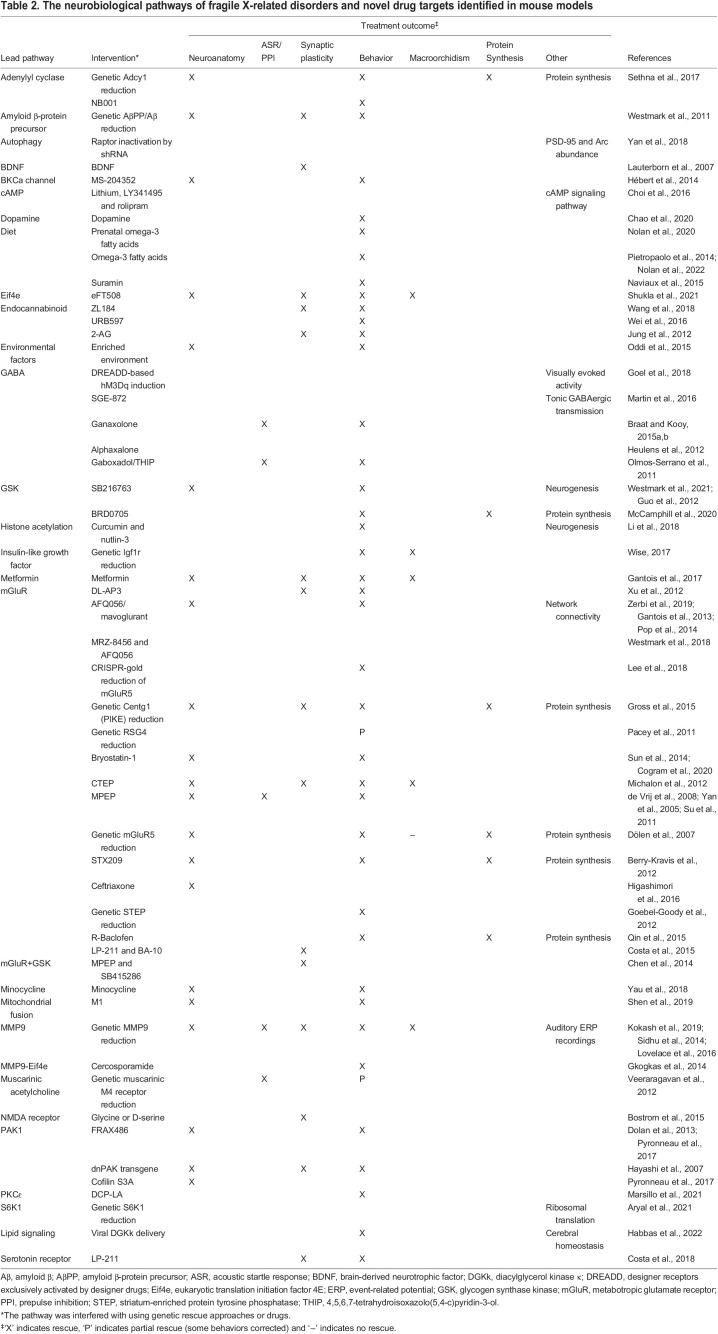
The neurobiological pathways of fragile X-related disorders and novel drug targets identified in mouse models

Although the number of drugs investigated in these studies appears overwhelming, these converge on a limited number of targets, including the mGluR pathway, the GABAergic and the dopaminergic systems, the serotonin receptor, autophagy and the amyloid β-protein precursor (see Table 2). Some of the studies have also been supported by genetic rescue experiments. For instance, many phenotypes of *Fmr1* KO mice have been rescued by treating these mice with drugs such as 2-chloro-4-({2,5-dimethyl-1-[4-(trifluoromethoxy)phenyl]-1H-imidazol-4-yl}ethynyl)pyridine (CTEP) that dampen mGLuR signaling (Michalon et al., 2012). Treatment in adult mice instantly corrected the elevated LTD and protein synthesis, as well as the audiogenic seizures. Chronic treatment rescued cognitive deficits, aberrant dendritic spine density and hyperactive ERK and mTOR signaling, and, in part, corrected macroorchidism. This finding has been complemented by the crossing of *Fmr1* KO mice with mice that are heterozygous for the gene that encodes the mGluR5 receptor (*Grm5*) (Dölen et al., 2007). Like the pharmacological rescue, the genetic cross restored multiple and widely diverse FXS phenotypes. Although the mGluR pathway has been the most extensively studied, encouraging results have been presented for pharmacological and/or genetic rescue of several other pathways involved in FXS (see Table 2 for an overview and the corresponding references).

### Clinical relevance of preclinical data

Encouraged by the preclinical findings (Table 2), a wide range of placebo-controlled clinical trials have been undertaken in recent years, each targeting a pathway demonstrated to be amenable to treatment in FXS mouse models (for an overview of these clinical trials, see Berry-Kravis et al., 2018; Jalnapurkar et al., 2019). Disappointingly, none of these interventions unambiguously demonstrated efficacy in clinical trials, even though some trials included large cohorts of patients. For instance, treating hundreds of patients with drugs that dampen mGluR signaling, such as basimglurant and mavoglurant (AFQ056), did not lead to any observed improvement in clinical presentation in the patient group (Berry-Kravis et al., 2016; Youssef et al., 2018). A critical note on the design of these clinical trials is warranted. With the exception of two (Berry-Kravis et al., 2016; Youssef et al., 2018), all other studies were statistically underpowered and some were even open label (reviewed in Berry-Kravis et al., 2018). Open-label trials in neurodevelopmental disorders are especially prone to biases as the placebo effect has been estimated at a bias-corrected standardized mean difference (Hedge's *g*) of 0.5, considered to be a mean effect size (Curie et al., 2015) and even higher at *g* 0.6 if the patients are certain of receiving the genuine medication as is the case in this type of trials (Jensen et al., 2017). Major limitations include, for instance, the age of treatment. Most studies recruited adolescents or adults and not young children, the patient group in which most benefits can be expected. In addition, the outcome measures of these clinical trials are far from optimal. In many trials, the primary outcome measure was a behavioral test, such as the aberrant behavior checklist or the Clinical Global Impression Improvement. These tests have been designed for clinical assessment, but not as outcome measures, as they lack the stability or sensitivity to track the efficacy of the intervention studied in the trial (Hessl et al., 2016; Shields et al., 2020).

Although trial design is likely to partly account for the lack of improvement observed in the clinical trials, the validity of the *Fmr1* KO mouse as a translational model needs to be critically appraised. At the time of writing this Review, we can only speculate about the reasons for the discrepancies between the human and murine studies, but a few factors could have played a role, such as (1) the use of inbred genetic mouse models – patients are as genetically heterogeneous as the general population, whereas the mouse lines used for FXS modeling are (almost) devoid of genetic variation; (2) subtle differences in mouse phenotypes and inconsistent results between laboratories (Crabbe et al., 1999; Kooy, 2003) – the differences between genotypes, in particular in behavioral tests, are usually subtle, hampering the reproducibility of the test (Dobkin et al., 2000; Paradee et al., 1999); (3) the outcome measures used to characterize the mice – these include behavioral deficits, the rate of protein synthesis, spine morphology, LTD or audiogenic seizures, which do not always correspond with human phenotypes; (4) the windows of plasticity, e.g. the neurodevelopmental period that is sensitive to treatment is unknown – we do not know to what extent sex, age and duration of treatment might influence the outcome of a clinical trial; and (5) the complex role of FMRP in neurons – targeting only a single pathway might not be sufficient to treat patients with an *FMR1* mutation and combination therapy might be necessary (van der Lei and Kooy, 2022; Zeidler et al., 2017).

## Key challenges and future perspectives

A key challenge for this field is to develop animal models that more closely recapitulate human FXS and related disorders, and to improve the reproducibility of the results derived from these models. To address these challenges, we suggest that preclinical studies should be carried out in different, preferably mixed, genetic backgrounds and that therapeutic efficacy should be reproduced by at least two independent laboratories before moving on to clinical trials. This would, in most cases, prevent over-reliance on incidental preclinical results. To further increase reproducibility, animal model experiments should be designed, performed and reported in accordance with the guidelines set out in the Planning Research and Experimental Procedures on Animals: Recommendations for Excellence (PREPARE) and Animal Research: Reporting of In Vivo Experiments (ARRIVE) 2.0 guidelines (Percie du Sert et al., 2020; Smith et al., 2018). In addition, new outcome measures in mice should be developed to be robust and translatable to humans, e.g. electroencephalogram recordings, functional magnetic resonance imaging, near-infrared spectroscopy and transcranial magnetic stimulation (Berry-Kravis et al., 2018; Zeidler et al., 2015). We also consider it important to report negative studies and that raw data, protocols and materials are made publicly accessible to address the publication bias in the reporting of positive results. The revised and updated ARRIVE 2.0 guidelines provide a checklist of recommendations to improve the reporting of research involving animals, thus maximizing the quality and reliability of published research, and enabling others to better scrutinize, evaluate and reproduce these studies (Percie du Sert et al., 2020).

Although such measures will likely increase the reproducibility of the results reported for FXS-related mouse models, they do not address the fundamental biological differences between humans and mice. Such limitations might be overcome by studying fragile X-related disorders in a range of model species. The *Drosophila dFmr1*-inactivated model, for example, has already been used to mass screen drugs that rescue the fly phenotype (Chang et al., 2008). Flies that express the CGG repeat only in the eye have been used to identify interactors and disease modifiers of the expanded CGG repeat (Jin et al., 2007; Sofola et al., 2007). The *fmr1* KO zebrafish also has the potential to be used for drug screening. High-throughput screening, however, requires a phenotype to present at a young age. This has only been observed in one study, in a CRISPR-generated 8-bp deletion KO zebrafish line (Hu et al., 2008), but not in a study that screened two N-ethyl-N-nitrosourea (ENU)-induced KO zebrafish lines, fmr1^hu2787^ and fmr1^hu2898^ (den Broeder et al., 2009). Other models to consider include non-human primates, which might facilitate investigations of FXS behavioral phenotypes (Curnow and Wang, 2022; Dahlhaus, 2018). However, the use of non-human primates in research has serious ethical implications, which limits their use in many countries, and is exceptionally costly. Such issues can be partly circumvented by the use of organoids, because some organoids can faithfully represent the complex, three-dimensional features of the human brain (Di Lullo and Kriegstein, 2017). They can also recapitulate typical human brain characteristics and organization, such as progenitor zone organization, outer radial glia cell layer organization and neurogenesis, and they can demonstrate similar gene expression profiles to human brains (Camp et al., 2015; Luo et al., 2016). So far, only two studies have described human forebrain organoids from FXS patient-derived induced pluripotent stem cells (Brighi et al., 2021; Kang et al., 2021). These FXS organoid forebrain models exhibited reduced proliferation of neural progenitor cells, as well as deregulated neural differentiation, increased synapse formation and neuronal hyperexcitability. The production of GABAergic neurons was also compromised. As such, these FXS organoids could provide a promising alternative platform for studying FXS in tissues that are otherwise inaccessible.

The combined generation of new animal and organoid models of fragile X-related disorders are likely to inform future translational studies that will hopefully result in new targeted treatments for FXS and FXTAS patients.
